# Universal screening for the SARS-CoV-2 virus on hospital admission in an area with low COVID-19 prevalence

**DOI:** 10.1017/ice.2020.358

**Published:** 2020-07-23

**Authors:** Sangeeta R. Sastry, Rachel Pryor, Jillian E. Raybould, Julie Reznicek, Kaila Cooper, Amie Patrick, Shelley Knowlson, Pamela Bailey, Emily Godbout, Michelle Doll, Michael P. Stevens, Gonzalo Bearman

**Affiliations:** Division of Infectious Diseases, Virginia Commonwealth University, Richmond, Virginia

Asymptomatic persons contribute to widespread transmission of the severe acute respiratory coronavirus virus 2 (SARS-CoV-2) and the coronavirus disease 2019 (COVID-19) pandemic.^1^ Published reports from areas of high COVID-19 incidence in the United States suggest that a significant percentage of asymptomatic persons are in healthcare systems. In 2 New York City (NYC) hospitals, 13.7% of asymptomatic pregnant women admitted for delivery tested positive for SARS-CoV-2 virus.^2^ Similarly, the nursing facility in Washington state with the earliest death from COVID-19 infection and the first healthcare worker infected in the United States, reported >50% positivity of their asymptomatic residents for the virus.^3^ Universal screening of healthcare populations may prevent in-hospital transmission of SARS-CoV-2 virus. However, testing resources and personal protective equipment (PPE) supplies to effectively isolate positive asymptomatic persons are currently limited, resulting in provider safety concerns. Upon developing real-time reverse-transcriptase polymerase chain reaction (rRT-PCR) tests in-house with >98% sensitivity, as well as increasing the availability of PPE at our institution, we initiated universal screening of patients on hospital admission using nasopharyngeal swabs to identify and isolate asymptomatic positive patients to prevent in-hospital transmission of SARS-CoV-2. We report our experience with universal screening of asymptomatic hospitalized persons, including a comparison of demographics between symptomatic and asymptomatic populations.

## Methods

On April 27, 2020, our 1,000-bed academic center instituted universal SARS-CoV-2 testing of patients on hospital admission. Clinicians performed COVID-19 symptom screening using clinical criteria reported in the literature.^4^ They designated patients as symptomatic or asymptomatic when ordering the test. An infectious diseases physician conducted chart review of asymptomatic positive patients to confirm accuracy of classification. Asymptomatic patients were not isolated; test turnaround time was 6–24 hours.

Statistical analyses were performed with the Fischer exact tests and paired *t* tests to compare asymptomatic and symptomatic positive patients using SAS version 9.4 software (SAS Institute, Cary, NC).

## Results

Between April 27, 2020, and May 18, 2020, when the hospital averaged at 60%–70% capacity, we performed 1,811 SARS-CoV-2 tests on nasopharyngeal specimens: 1,335 (74%) were asymptomatic, 420 (23%) were symptomatic, 56 (3%) were incorrectly ordered. Of the 1,755 tests in this analysis, overall positivity for SAR-CoV-2 virus was 79 (4.5%). Of 79 patients, 12 were asymptomatic (15%) and 67 were symptomatic (85%). Of 1,335 asymptomatic patients, 12 tested positive, for a rate of ~ 1%. Of 420 symptomatic patients, 67 tested positive, for a rate of 16%. No test converted to positive among asymptomatic patients while hospitalized.

A comparative analysis of patients with positive SARS-CoV2 tests is listed in Table 1. The mean age of asymptomatic patients was 37 years (SD, 19.71) versus a mean age of 59 years (SD, 13.08) among symptomatic patients (*P =* .0020). Hispanic patients were more likely to be asymptomatic (7 of 12) than symptomatic (9 of 67) at the time of testing (58% vs 13%; *P* = .0017). We observed no difference in positivity rate on admission of asymptomatic versus symptomatic patients (*P* = .21). In addition, 5 asymptomatic positive women were pregnant (5 of 12, 42%); no symptomatic patients were pregnant (*P ≤* .0001). A baby born to an asymptomatic SARS-CoV-2–positive mother tested positive at 48 hours of life, and 1 asymptomatic, SARS-CoV-2–positive, immunocompromised patient was receiving chemotherapy for breast cancer. One asymptomatic patient developed a fever during hospitalization, and another was readmitted within 14 days of testing positive, both of these events were not considered to be related to COVID-19.

**Table 1.** Comparative Analysis Between Asymptomatic and Symptomatic Patients With Positive SARS-CoV-2 Virus Tests

Demographic Characteristics of Asymptomatic Patients, SARS-CoV-2–Positive Patients (N = 12, 0.62%)		Demographic Characteristics of Symptomatic Patients, SARS-CoV-2–Positive Patients (N = 67, 3.5%)		*P* Value
**Mean age, y (range)**	37 (0–67) (SD, 19.71)	**Mean age, y (range)**	59 (12–78) (SD, 13.08)	.0020
	No. (%)		No. (%)	
**Race/ethnicity**		**Race/ethnicity, y (range)**		
Hispanic	7 (58)	Hispanic	9 (13)	.0017
African American	4 (33)	African American	36 (54)	.22
Caucasian	1 (8)	Caucasian	14 (21)	.44
Other	0 (0)	Other	8 (12)	
**Gender**		**Gender**		
Male	5 (42)	Male	42 (63)	.21
Female	7 (58)	Female	25 (37)	
Pregnant	5/7 (42)	Pregnant	0/25 (0)	≤.0001

Note. SD, standard deviation.

## Discussion

Universal screening for the detection of SARS-CoV-2 at our institution revealed that during the study period, the number of asymptomatic persons admitted to the hospital was relatively small. Our health system had a relatively low number of confirmed SARS-CoV-2–positive COVID-19 patients (n = 82) admitted during the observed 3-week interval, compared to 4,000 patients admitted to an NYC hospital reporting the use of convalescent serum for the treatment of COVID-19 in a similar time frame.^5^ Although low prevalence of asymptomatic patients has limited generalizability to areas with higher rates of infection, it is valuable information for patients, healthcare workers, and epidemiology programs in similar areas of COVID-19 prevalence.

During our study period, 7.6% of all admitted patients were Hispanic and 43.5% were African American, yet 11 of 12 (91.7%) asymptomatic patients who screened positive were African American or Hispanic. A similar trend was observed in other studies.^6,7^ Furthermore, a higher proportion of pregnant women have asymptomatic infection, which supports screening of peripartum women. Consistent with the literature, asymptomatic patients were younger than those who presented to our healthcare system with COVID-19 symptoms.^8^

The potential benefits of universal SARS-CoV-2 screening are many and are likely to increase with escalating COVID-19 incidence. In hospitalized patients, detection of asymptomatic infection can guide hospital isolation practices, bed assignments, and the use of PPE.^2^ For healthcare workers, it might improve workforce depletion by unnecessary quarantine, reduce transmission in asymptomatic cases, contain the virus in healthcare settings, and protect hospital staff from infection. In the community, mass testing can identify asymptomatic cases and assist in eliminating the SARS-CoV-2 virus, as reported in a village near Venice, Italy.^9^

However, there are barriers to universal screening. Current testing capacity and test turnaround time, staffing shortages, and availability of healthcare workers skilled to perform nasopharyngeal swabbing currently limit widespread feasibility. Patient discomfort from nasopharyngeal sample collection is another potential barrier to universal screening.

This study has several limitations. The sample size was small, and the study was conducted at a single center. In an area with high prevalence of COVID-19 infection, asymptomatic screening would likely identify more asymptomatic cases. However, sensitivity of a test in asymptomatic persons cannot be precisely defined. We add to the body of literature on SARS-CoV-2 testing of asymptomatic patients at the time of hospital admission. More data on universal screening is necessary to evaluate the clinical impact on healthcare systems and to define optimal screening strategies of high-risk groups for asymptomatic COVID-19 infection.
